# Restoration of FMRP expression in adult V1 neurons rescues visual deficits in a mouse model of fragile X syndrome

**DOI:** 10.1007/s13238-021-00878-z

**Published:** 2021-10-29

**Authors:** Chaojuan Yang, Yonglu Tian, Feng Su, Yangzhen Wang, Mengna Liu, Hongyi Wang, Yaxuan Cui, Peijiang Yuan, Xiangning Li, Anan Li, Hui Gong, Qingming Luo, Desheng Zhu, Peng Cao, Yunbo Liu, Xunli Wang, Min-hua Luo, Fuqiang Xu, Wei Xiong, Liecheng Wang, Xiang-yao Li, Chen Zhang

**Affiliations:** 1 State Key Laboratory of Membrane Biology, PKU-IDG/McGovern Institute for Brain Research, School of Life Sciences, Peking University, Beijing, 100871 China; 2 School of Basic Medical Sciences, Beijing Key Laboratory of Neural Regeneration and Repair, Capital Medical University, Beijing, 100069 China; 3 Peking-Tsinghua Center for Life Sciences, Academy for Advanced Interdisciplinary Studies, Peking University, Beijing, 100871 China; 4 School of Mechanical Engineering and Automation, Beihang University, Beijing, 100191 China; 5 School of Life Sciences, Tsinghua University, Beijing, 100084 China; 6 Britton Chance Center for Biomedical Photonics, Wuhan National Laboratory for Optoelectronics-Huazhong University of Science and Technology, Wuhan, 430074 China; 7 State Key Laboratory of Brain and Cognitive Sciences, Institute of Biophysics, Chinese Academy of Sciences (CAS), Beijing, 100101 China; 8 Institute of Laboratory Animal Science, Peking Union Medical College/Chinese Academy of Medical Science, Beijing, 100021 China; 9 Laboratory Animal Center, Fujian University of Tradition Chinese Medicine, Fuzhou, 350122 China; 10 State Key Laboratory of Virology, CAS Center for Excellence in Brain Science and, Intelligence Technology, Wuhan Institute of Virology, CAS, Wuhan, 430071 China; 11 Center for Brain Science, Key Laboratory of Magnetic Resonance in Biological Systems and State Key Laboratory of Magnetic Resonance and Atomic and Molecular Physics, Wuhan Institute of Physics and Mathematics, CAS, Center for Excellence in Brain Science and Intelligence Technology, Chinese Academy of Sciences, Wuhan, 430071 China; 12 Department of Physiology, Anhui Medical University, Hefei, 230032 China; 13 Department of Physiology, Institute of Neuroscience and Collaborative Innovation Center for Brain Science, School of Medicine, Zhejiang University, Hangzhou, 310058 China

**Keywords:** autism spectrum disorder, calcium imaging, fragile X syndrome, primary visual cortex, visual hypersensitivity

## Abstract

Many people affected by fragile X syndrome (FXS) and autism spectrum disorders have sensory processing deficits, such as hypersensitivity to auditory, tactile, and visual stimuli. Like FXS in humans, loss of *Fmr1* in rodents also cause sensory, behavioral, and cognitive deficits. However, the neural mechanisms underlying sensory impairment, especially vision impairment, remain unclear. It remains elusive whether the visual processing deficits originate from corrupted inputs, impaired perception in the primary sensory cortex, or altered integration in the higher cortex, and there is no effective treatment. In this study, we used a genetic knockout mouse model (*Fmr1*^KO^), *in vivo* imaging, and behavioral measurements to show that the loss of *Fmr1* impaired signal processing in the primary visual cortex (V1). Specifically, *Fmr1*^KO^ mice showed enhanced responses to low-intensity stimuli but normal responses to high-intensity stimuli. This abnormality was accompanied by enhancements in local network connectivity in V1 microcircuits and increased dendritic complexity of V1 neurons. These effects were ameliorated by the acute application of GABA_A_ receptor activators, which enhanced the activity of inhibitory neurons, or by reintroducing *Fmr1* gene expression in knockout V1 neurons in both juvenile and young-adult mice. Overall, V1 plays an important role in the visual abnormalities of *Fmr1*^KO^ mice and it could be possible to rescue the sensory disturbances in developed FXS and autism patients.

**Supplementary Information:**

The online version contains supplementary material available at 10.1007/s13238-021-00878-z.

## Introduction

Sensory processing abnormalities in fragile X syndrome (FXS) and other autism-related disorders seriously affect people’s lives in the form of hyperactivity, anxiety, and communication and cognitive difficulties. Hypersensitivity to sensory stimuli is a prominent feature of both FXS and autism spectrum disorder (ASD) (Sinclair et al., 2017) and has been seen in many different brain areas, such as the auditory cortex, somatosensory cortex, and visual cortex (Molen et al., 2012a; Molen et al., 2012b; Rais et al., 2018). In FXS and ASD patients, abnormalities in low-level visual perception and processing have been linked to impaired recognition of facial expression and social interaction (Dakin and Frith 2005). The sensory deficits in FXS are extensive. FXS patients exhibit abnormal responses, including both hypo- and hyper-sensitivity, to external stimuli such as sound, touch, or visual avoidance (Molen et al., 2012a; Molen et al., 2012b; Rais et al., 2018). However, the neurological mechanisms of sensory abnormalities in FXS and ASD, especially visual impairment, remain unclear.

 The *Fmr1*^KO^ mouse model is an ideal animal model to study this question, as these mice show similar sensory deficits to those of FXS patients and exhibit autistic-like behaviors, including repetitive behaviors, hyperactivity, and seizures (Musumeci et al., 2000). In murine models, loss of FMR1 caused molecular, cellular, and functional defects in the retina (Rossignol 2014), leading to immature retinal neuron development and abnormal dendritic morphology, both of which appeared before eye opening. Studies in juvenile *Fmr1* KO rats also showed abnormal visual responses to simple luminance stimuli (Berzhanskaya et al., 2016). Mice lacking the *Fmr1* gene displayed abnormal ocular dominance plasticity of the visual cortex during development, which indicates that experience-dependent synaptic modification is altered (Dölen et al., 2007). Vision integration is particularly affected in *Fmr1*^KO^ mice along with altered spatiotemporal visual processing and orientation-tuning sensitivity for visual stimuli (Goel et al., 2018). These abnormalities are considered to be correlated with cortical immaturity, especially in the primary visual cortex (V1) (Berman et al., 2012). *Fmr1*^KO^ mice showed impaired visuospatial discrimination, which is associated with decreases in synaptic proteins in the prefrontal cortex (PFC) (Sidorov et al., 2014).

Most studies on visual abnormalities of FXS and ASD focus on the neuroimaging and behavior levels (Churchill et al., 2002; Farzin et al., 2008; Pereira et al., 2018). Both behavior and functional MRI experiments have shown that reduced global motion perception in autism is reflected as early as the primary visual cortex (Robertson et al., 2014). In addition, when measuring first- and second-order information processing along the parvocellular pathway, researchers found high-functioning autists are superior at identifying the orientation of simple, first-order gratings, which are processed in V1, while inferior at identifying the orientation of second-order gratings when compared with normal controls (Armando et al., 2005). However, little is known about the cellular and neural circuit mechanisms underlying specific behaviors of visual abnormalities. The study of single-gene inherited diseases, such as FXS, can provide opportunities for understanding the causes of ASD.

Here, we try to find the mechanism of vision impairment in FXS along with a therapeutic strategy. We investigated the neural mechanisms underlying visual perception in conscious, head-fixed *Fmr1*^KO^ mice by combining *in vivo* two-photon imaging with fidget behavior measurements and genetic approaches. Our results demonstrate that the deletion of *Fmr1* in V1 neurons is necessary and sufficient to cause abnormal visual sensitivity and that this cell-autonomous deficit could be rescued either by acutely boosting GABA receptor function or by locally expressing FMR1 in developing V1 microcircuits. Thus, our data suggest that V1 is the key brain region responsible for the visual abnormalities in FXS mice, which provides a possible way to rescue the sensory disturbances observed in FXS and autism patients.

## Results

### ***Fmr1***^KO^ mice exhibit enhanced sensitivity to low-intensity visual stimulation

**Figure 1:**
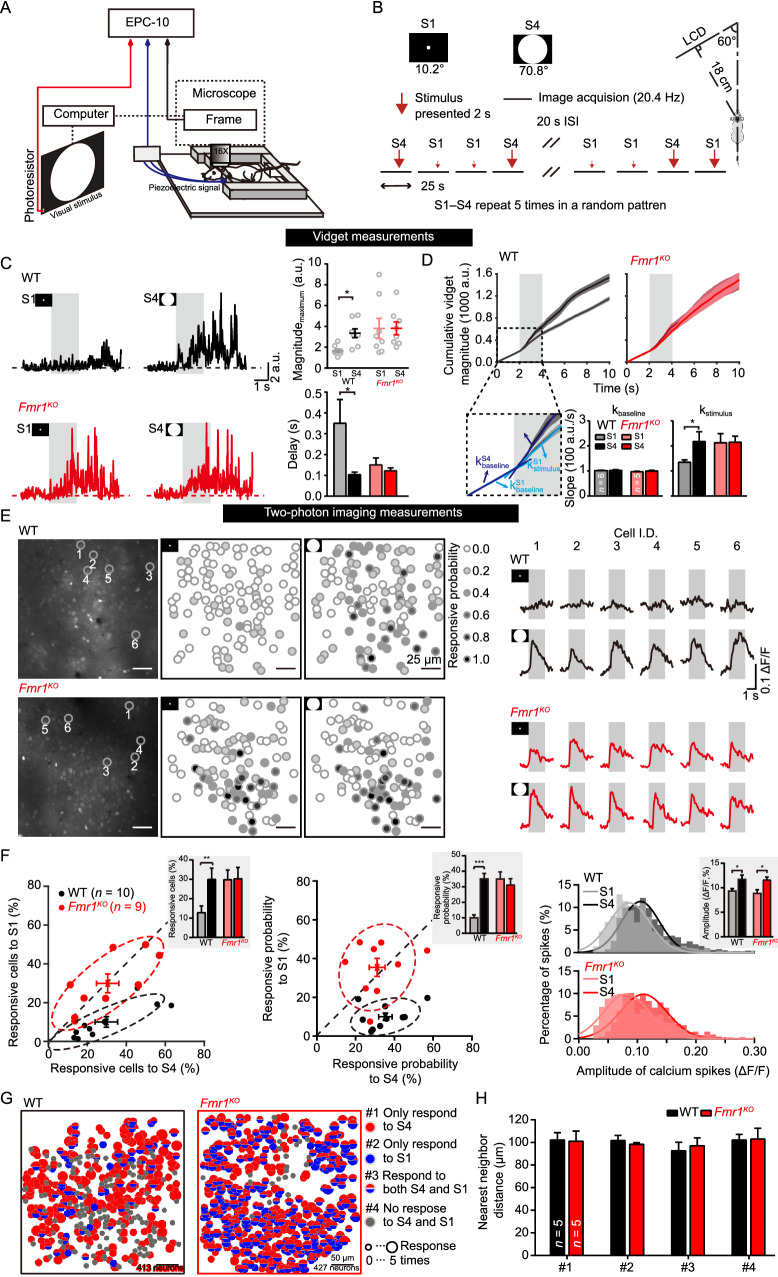
Enhanced visual sensitivity in *Fmr1*^*KO*^ mice. (A) Experimental setup for two-photon imaging and behavioral experiments. A piezoelectric sensor was placed under the forepaws of the mice and all signals from various inputs were sent to an EPC10 amplifier for synchronization. (B) Diagram of the stimulus strategy. The visual stimuli S1 and S4 represent 10.2° and 70.8° of the visual field (Φ_S4_ = 8*Φ_S1_), respectively. S1 and S4 were repeated 5 times in a random pattern. Each stimulus was presented for 2 s, and the stimuli were separated by a 20-s black screen. (C) Behavioral responses of wild-type (WT) and *Fmr1*^KO^ mice to S1 and S4. Left, average traces of five visual stimulus-induced fidget responses in WT (black) and *Fmr1*^KO^ (red) mice measured by piezoelectric voltage signals. Gray regions indicate the periods of visual stimulation. Right, summary graph of vidget magnitude (arbitrary units, (a.u.), WT: S1: 1.64 ± 0.17, S4: 3.34 ± 0.42, *P* < 0.01; *Fmr1*^KO^: S1: 3.81 ± 0.97, S4: 3.81 ± 0.62, *P* > 0.05) and the delay (WT, S1: 0.35 ± 0.11 s, S4: 0.10 ± 0.01 s, *P* < 0.05; *Fmr1*^KO^: S1: 0.15 ± 0.03 s, S4: 0.12 ± 0.01 s, *P* > 0.05) in behavioral response. The calculation of the delay for vidget response is shown in Fig. S1. (D) Cumulative vidget magnitude in WT (black) and *Fmr1*^KO^ (red) mice. Above: The implementation of piecewise linear fitting and the definition of the slopes for WT and *Fmr1*^KO^ mice. Bottom: summary graph of the slopes for WT (${\mathrm{k}}_{\mathrm{baseline}}$ (1,000 a.u./s), S1: 1.02 ± 0.01, S4: 1.02 ± 0.03, *P* > 0.05; ${\mathrm{k}}_{\mathrm{stimulus}}$, S1: 1.35 ± 0.09, S4: 2.17 ± 0.39, *P* < 0.05) and *Fmr1*^KO^ (${\mathrm{k}}_{\mathrm{baseline}}$, S1: 0.97 ± 0.02, S4: 1.00 ± 0.03, *P* > 0.05; ${\mathrm{k}}_{\mathrm{stimulus}}$, S1: 2.13 ± 0.36, S4: 2.15 ± 0.24, *P* > 0.05) mice. Gray regions indicate periods of visual stimulation. (E) Representative view of two-photon imaging and the calcium signal of V1 neurons. Left, Representative images of V1 obtained using an *in vivo* two-photon calcium imaging technique and the response probability of neurons to S1 and S4. The white circle indicates a neuron that had no response to the visual stimulus. The black circle indicates a neuron that responded to the visual stimulus every time. Right panel, average calcium signals obtained in 5 trials of the labeled neurons. The gray area denotes the stimulus period (2 s). The trace included a 1-s prestimulus period, 2 s of stimulus presentation (gray region), and a 1-s poststimulus period. (F) Analyses of the cell responds to S1 and S4. Left: Scatter plot of the average percentage of cells that were responsive to S1 versus S4 in WT (black) and *Fmr1*^KO^ (red) mice. Inset panel, summary graph of the average percentage (WT, S1: 10.2% ± 2.8%, S4: 29.8% ± 5.8%, *P* < 0.01; *Fmr1*^KO^, S1: 30.1% ± 5.0%, S4: 30.2% ± 5.8%, *P* > 0.05) of responsive cells. Middle: Scatter plot of the average probability of cells being responsive to S1 versus S4 in WT (black) and *Fmr1*^KO^ (red) mice. Inset panel, summary graph of the average probability (WT: S1: 0.10 ± 0.02, S4: 0.35 ± 0.03, *P* < 0.001; *Fmr1*^KO^, S1: 0.35 ± 0.05, S4: 0.31 ± 0.04, *P* > 0.05) of a response. Right: Statistics for the amplitudes of calcium spikes in WT (black) and *Fmr1*^KO^ (red) mice in response to S1 and S4. Inset panel, summary graph of the amplitudes (WT, S1: 0.093 ± 0.005, S4: 0.117 ± 0.009, *P* < 0.05; *Fmr1*^KO^, S1: 0.088 ± 0.008, S4: 0.115 ± 0.006, *P* < 0.05). (G) Four functional classes of neurons classified according to neuronal responses to S1 and S4. Locations and types of neurons responding to S1 and S4 in WT and *Fmr1*^KO^ mice. Type #1 (red circles) neurons responded specifically to S4. Type #2 (blue circles) neurons responded specifically to S1. Type #3 (semired and semiblue circles), neurons responded to both S4 and S1. Type #4 (gray circles) neurons did not respond to either stimulus. The radius of each circle or semicircle is linearly correlated with the probability of a response to S1 or S4. (H) Summary graph of the nearest neighbor distance (NND)-based distance for four types of neurons (#1, WT: 102.11 ± 6.50, *Fmr1*^KO^:101.10 ± 8.94, *P* > 0.05; #2, WT: 101.68 ± 4.60, *Fmr1*^KO^: 98.39 ± 1.48, *P* > 0.05;#3, WT: 92.60 ± 7.60, *Fmr1*^KO^: 97.04 ± 7.00, *P* > 0.05; #4, WT: 102.15 ± 4.96, *Fmr1*^KO^: 103.05 ± 9.52, *P* > 0.05). NND-based distance indicating the degree of neuronal spatial distribution as calculated from randomly selected k-subgroups (each k-subgroup contained k neurons, k = 5). Data are shown as the mean ± s.e.m. Kruskal-Wallis test *P < 0.05, **P < 0.01, ***P < 0.001

To detect whether inactivation of *Fmr1* gene expression affects the animal's visual perception and investigate the response to visual stimuli at the single-neuron level in *Fmr1*^KO^ mice, we performed an *in vivo* two-photon experiment in conscious mice. The animals’ behavioral responses to the visual stimuli were evaluated using visually induced fidget (vidget) measurements from a piezoelectric sensor placed under the forepaws of the animal (Cooke et al., 2015; Tian et al., 2018). The precise timing of the visual stimulus was determined via simultaneous photoresistance recording, and all signals from various inputs were sent to an EPC10 amplifier for synchronization (Fig. 1A).

We examined the input-output (vidget behavior) relationship with regard to the intensity of the visual stimulus. We modulated the intensity of the visual stimuli by presenting white spots of different sizes (on a black background) at the center of an LCD screen (Fig. 1B). Compared to the low-intensity stimulus S1, the high-intensity visual stimulus S4 induced a significantly higher vidget response in wild-type (WT) mice, as reflected by its higher magnitude and shorter delay. However, in *Fmr1*^KO^ mice, the same two stimuli elicited indistinguishable vidget responses in terms of both magnitude and delay (Figs. 1C and S1). We implemented piecewise linear fitting to determine the cumulative vidget magnitude during the baseline and stimulation periods. The slope of the baseline period indicated that there were no significant differences between the responses for either the two stimuli or the two genotypes (Fig. 1D). Consistent with these results, the WT mice exhibited a significantly higher slope in response to the high-intensity stimulus than the low-intensity stimulus, while in *Fmr1*^KO^ mice, there was no significant difference between the slopes for the two stimuli. These results clearly demonstrate an altered input-output relationship in the visual perception of stimulus intensity between genotypes.

Next, we monitored the activities of neurons labeled with the calcium indicator Oregon Green 488 BAPTA-1-AM (OGB-1) in V1. In WT mice, the percentage of responsive V1 neurons increased from 10.2% ± 2.8% to 29.8% ± 5.8% when the diameter of the stimulus increased from 10.2° (S1) to 70.8° (S4) (Fig. 1E and 1F, left). In contrast, no difference in the percentage of S1-activated and S4-activated neurons was observed in *Fmr1*^KO^ mice, but the percentage of S1-responsive V1 neurons was significantly higher in *Fmr1*^KO^ mice than in WT mice (Fig. 1E and 1F, left). We further analyzed the probability of cell responsiveness to the stimuli. V1 neurons in WT mice exhibited a significantly higher probability of responding to S4 than to S1 (Fig. 1F, middle). However, V1 neurons in *Fmr1*^KO^ mice exhibited almost the same probability of response to the two stimuli. The amplitude of calcium spikes elicited by the visual stimuli followed normal distributions in both genotypes (Fig. 1F, right, Gaussian fitting), and both WT and *Fmr1*^KO^ mice showed higher amplitudes of calcium spikes to S4 than S1. No significant differences in amplitude were observed between WT and *Fmr1*^KO^ mice in response to either stimulus (Fig. 1F, right, inset). Additionally, the average visual-stimulus-induced neuronal and behavioral responses of the *Fmr1*^KO^ mice in trials in which S1/2/3/4 were presented in a random pattern differed from those of the WT mice (Fig. S2). Thus, *Fmr1*^KO^ mice show hypersensitivity to low-intensity stimulus at the behavioral and cellular level.

To examine whether responsive neurons show spatial preferences, or in other words whether these cells are clustered in space, we adopted a nearest neighbor distance (NND) (Zhang et al., 2006)-based method to quantify the degree of cell distribution in space. To avoid interference by cell number, we applied a random k-subgroup offset to the NND method (reliability test in Fig. S3). All recorded neurons were classified into four groups: type #1 and #2 neurons responded specifically to S4 and S1, respectively; type #3 neurons responded to both stimuli; and type #4 neurons did not respond to either stimulus (Fig. 1G). We found that the degree of neuronal distribution in space was not significantly different among cell types or genotypes (Fig. 1H, k = 5, where k indicates a randomly selected k-subgroup that contained k neurons, Fig. S4, k = 5–10). As the distribution of neurons in the adult cortex is the result of a complex interaction of developmental/evolutionary determinants and functional interactions (Atapour, et al., 2018), this result indicates that the loss of FMR1 does not implicate neuronal development in the young adult mouse V1.

Together, these data indicate that the inactivation of the *Fmr1* gene in mice specifically increased the responsiveness of cells to weaker visual stimuli but not to high-intensity stimuli, as reflected by both V1 neuronal activity and the concomitant vidget behavior.

### *Fmr1* deletion enhances functional connectivity in the V1 local circuit

**Figure 2:**
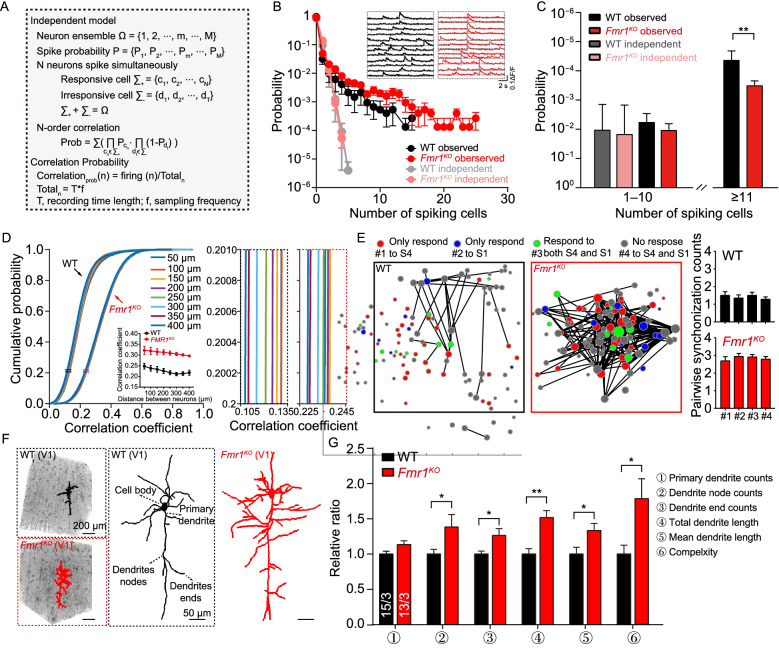
Enhanced functional local connections and dendritic complexity of V1 neurons in *Fmr1*^KO^ mice. (A) Calculation of the correlation probability for synchronous spiking events. The *n*-order correlation indicates the probability that *n* neurons fire spikes simultaneously. The independent model assumes that all neurons in the circuit are uncorrelated. The spike probability sequence was determined from spontaneous recordings. (B) Observed probability distribution and representative traces (inset) of synchronous spiking events obtained from spontaneous recordings of V1 neurons in WT (black) and *Fmr1*^KO^ (red) mice. The independent probability was calculated by randomly shuffling the spiking events 10,000 times (WT: gray; *Fmr1*^KO^: light red). (C) Statistics comparing the probability of the synchronization of spiking cells between WT and *Fmr1*^KO^ mice (probability of synchronization (1–10), WT: 0.0251 ± 0.0097, *Fmr1*^KO^: 0.0246 ± 0.0094, *P* > 0.05; probability of synchronization (11–∞), WT: (4.4 ± 2.3) × 10^−5^, *Fmr1*^KO^: (3.3 ± 1.1) × 10^−4^, *P* < 0.01). (D) Cumulative probability of correlation coefficients versus the distances between neuronal pairs obtained from spontaneous recordings of V1 neurons in WT and *Fmr1*^KO^ mice. In the inset panel, the mean correlation coefficient is shown versus the distance between neuronal pairs. Right, partial enlargement of the correlation coefficients. (E) Synchronization of four functional assemblies (#1, #2, #3, and #4). Pairwise synchronization counts were calculated according to 73.5-s two-photon recordings of spontaneous activity (sampling frequency, 20.4 Hz; 1,500 frames in total). Neural assembly was determined according to responses to visual stimuli (Fig. 1H). Left, image plot of pairwise synchronization counts for four functional assemblies (synchronization counts: WT, 1.32 ± 0.0.13, *Fmr1*^KO^: 2.82 ± 0.13, *P* < 0.001). Right, summary plot of intersynchronization counts for each functional assembly. (F) 3D and 2D plots of the reconstructed morphologies of V1 neurons. (G) Summary graph of the relative proportions of primary dendrite counts using micro-optical sectioning tomography technology (WT: 1.00 ± 0.16, *Fmr1*^KO^: 1.13 ± 0.20, *P* > 0.05), dendrite node counts (WT: 1.00 ± 0.30, *Fmr1*^KO^: 1.52 ± 0.36, *P* < 0.05), dendrite end counts (WT: 1.00 ± 0.37, *Fmr1*^KO^: 1.33 ± 0.36, *P* < 0.05), total dendrite length (WT: 1.00 ± 0.26, *Fmr1*^KO^: 1.38 ± 0.64, *P* < 0.05), mean dendrite length (WT: 1.00 ± 0.16, *Fmr1*^KO^: 1.38 ± 0.64, *P* < 0.05), and dendrite complexity (WT: 1.00 ± 0.47, *Fmr1*^KO^: 1.79 ± 0.99, *P* < 0.05). All six measurements are shown normalized by the corresponding average value obtained in WT mice. Data are shown as the mean ± s.e.m. *P < 0.05, **P < 0.01, ***P < 0.001

To investigate the mechanism underlying this visual processing abnormality at the circuit level, we quantitatively measured the connectivity strength of the neural microcircuits in V1 by calculating the correlation efficiency as previously described (Tian et al., 2018). We calculated the probability of simultaneous calcium spikes occurring in *n* neurons (*n*-order correlation) and the probability of an *n*-order correlation. Compared with the independent model (Fig. 2A), both WT and *Fmr1*^KO^ mice showed a significantly higher percentage of synchronous firing, in the range of 5 or more per cell in a 40-neuron population (Fig. 2B). We then compared the probability of simultaneous calcium spikes occurring in neurons and found that the *Fmr1*^KO^ mice had more high-order synchronous spikes than the WT mice (Fig. 2C, Probability of Synchronization, (1–10): *P* > 0.05; (11–∞): *P* < 0.01). Thus, the connectivity of the neural microcircuits in V1 was enhanced in *Fmr1*^KO^ mice.

The mature cortical network is characterized by desynchronization and lacks a clear spatial structure. Having demonstrated that *Fmr1*^KO^ mice produce more high-order synchronous spikes, we further analyzed the pairwise coefficients and spatial structure of spontaneous neuronal activity according to the distance between neurons as before (Golshani et al., 2009). We calculated the Euclidean distance between each pair of neurons and grouped the cell pairs according to their relative distance. Compared with WT mice, *Fmr1*^KO^ mice demonstrated significantly higher correlation coefficients at all relative distances tested (Fig. 2D, two-way ANOVA, *P* < 0.001). We combined spontaneous activity and visual stimulus-induced activity to investigate whether the increased synchronization observed in *Fmr1*^KO^ mice was functional-assembly-specific. We presented all neurons in each group using circles whose diameters were proportional to synchronization counts. The neuronal pairs in *Fmr1*^KO^ mice showed stronger synchronization than those in WT mice (synchronization counts: WT, 1.32 ± 0.13, *Fmr1*^KO^: 2.82 ± 0.13, *P* < 0.001); however, the four types of neuronal assembly showed no significant differences in pairwise synchronization counts for either the WT or *Fmr1*^KO^ mice (Fig. 2E). These observations demonstrate that *Fmr1*^KO^ mice displayed abnormal desynchronization but a normal spatial structure in V1, which could explain the abnormalities in visual processing in *Fmr1*^KO^ mice.

To study whether enhanced local connectivity was accompanied by a change in structure, we examined the morphological complexity of *Fmr1*^KO^ V1 neurons using micro-optical sectioning tomography (MOST) (Li, et al., 2010). *Fmr1*^KO^ V1 neurons exhibited comprehensive structural alterations, including a significant increase in the numbers of dendrite nodes and dendrite ends and the magnitude of total and mean dendrite lengths (Fig. 2F and 2G). Moreover, a change in dendritic complexity (DC) was not observed in neurons in the lateral geniculate nucleus (LGN, Fig. S5A–C), which sends projections to V1. As a control, neurons in PFC regions were evaluated and found to be unaltered (Fig. S5D–F), suggesting that the observed increase in DC was likely specific to V1 neurons. Thus, an increased DC within V1 in *Fmr1*^KO^ mice provides a plausible explanation for the enhanced correlated activity observed in its neuronal populations.

### Conditional deletion of *Fmr1* in V1 neurons is sufficient to induce enhanced visual responsiveness and high local connectivity

**Figure 3:**
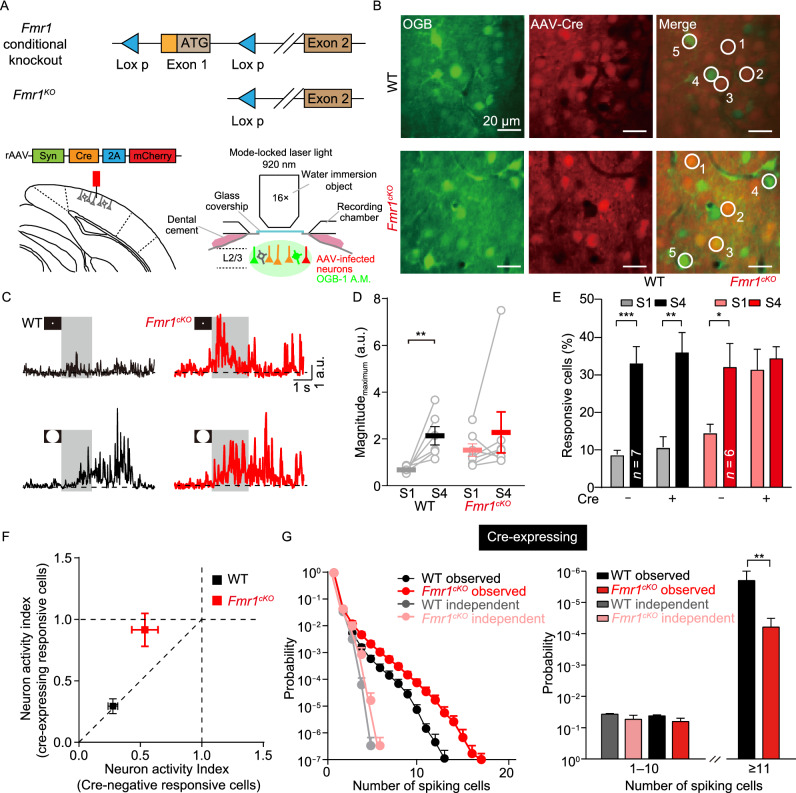
Conditional inactivation of FMR1 specifically in V1 neurons causes enhanced neural responses to weak (S1) but not strong (S4) stimuli. (A) Strategy for selective knockout of the *Fmr1* gene in V1 neurons using adeno-associated viruses (AAVs) in *Fmr1*^cKO^ mice. Bottom left, AAVs locally injected in V1. Bottom right, schematic representation of two-photon imaging experiment. (B) Illustrative fluorescence images obtained at layer 2/3 (L2/3) in the V1 of WT and *Fmr1*^cKO^ mice infected with AAV-Cre-mCherry. Green, OGB-1; red, mCherry; bar: 20 μm. The white circles indicate the OGB-1-labeled neurons. Neurons 1–3 were infected with AAV-Cre-mCherry. Adjacent neurons 4 and 5 were not infected and were used as internal controls. (C) Behavioral responses (vidgets) of WT and *Fmr1*^cKO^ mice to S1 and S4 in arbitrary units (a.u.). Grey bars represent the duration of visual stimulation. (D) Summary graph of vidget magnitude (in a.u.): WT: S1: 0.68 ± 0.06, S4: 2.14 ± 0.39, *P* < 0.01; *Fmr1*^cKO^: S1: 1.52 ± 0.27, S4: 2.27 ± 0.87, *P* > 0.05). (E) Summary graph of the average percentages (five trials, with each trial containing S1 and S4 in a random pattern) of responsive AAV-uninfected (Cre^-^) and AAV-infected (Cre^+^) neurons in V1 in WT (Cre^-^, S1: 8.8% ± 1.1%, S4: 33.4% ± 4.6%, *P* < 0.001; Cre^+^, S1: 10.7% ± 2.8%, S4: 36.4% ± 5.3%, *P* < 0.01) and *Fmr1*^cKO^ (Cre^-^, S1: 14.7% ± 2.2%, S4: 32.4% ± 6.3% *P* < 0.05; Cre^+^, S1: 31.7% ± 5.5%, S4: 34.7% ± 3.2%, *P* > 0.05) mice (AAV injection performed at P35). (F) Plots showing changes in the neuronal activity index (NAI) in Cre-infected and Cre-uninfected neurons in WT and *Fmr1*^cKO^ mice. (G) Left, probability distribution of synchronous spiking events occurring in spontaneous activities obtained in Cre-infected V1 neurons in WT (black) and *Fmr1*^cKO^ (red) mice. The independent probability was calculated by randomly shuffling the spiking events 10,000 times (WT: gray; *Fmr1*^cKO^: light red). Right, statistics comparing the probability of the synchronization of spiking cells between WT and *Fmr1*^KO^ mice (probability of synchronization (1–10), WT: 0.042 ± 0.0017, *Fmr1*^cKO^: 0.063 ± 0.0094, *P* > 0.05; probability of synchronization (11–∞), WT: (2.4 ± 1.3) × 10^−6^, *Fmr1*^*cKO*^: (6.5 ± 2.9) × 10^−5^, *P* < 0.01). Data are shown as the mean ± s.e.m. *P < 0.05, **P < 0.01, ***P < 0.001

To examine the precise brain region that causes the visual processing abnormalities observed in *Fmr1*^KO^ mice, we selectively inactivated *Fmr1* expression in V1 neurons by stereotactically injecting a Cre-expressing adeno-associated virus (AAV) (AAV9-hsyn-cre-mCherry-WPRE-pA) into V1 neurons in *Fmr1*^cKO^ mice at postnatal day 35 (P35), and monitored the neuron activities using two-photon imaging (Fig. 3A). The infected neurons were identified via the mCherry signal, which does not overlap with the calcium indicator OGB-1 in its excitation/emission spectrum. Using this approach, we monitored the activity in both WT (which lacked Cre and were OGB-positive; e.g., cell nos. 4–5 in Fig. 3B, top) and *Fmr1*^cKO^ (which expressed Cre and were OGB-positive, e.g., cell nos. 1–3 in Fig. 3B, bottom) neurons and thus examined the effect of cell-autonomous inactivation of *Fmr1* on V1 neurons while keeping the top-down and bottom-up pathways to the V1 region intact. In a control experiment, overexpression of Cre recombinase in the V1 region of WT mice did not alter visually induced behavioral responses (Fig. 3C and 3D, left) or intensity-dependent neural responses (Fig. 3E). In contrast, we found that conditional inactivation of *Fmr1* in V1 neurons caused significant increases in the magnitude of the behavioral response to S1 (Fig. 3C and 3D, right) and the number of neurons activated by low-intensity stimuli but not high-intensity stimuli (Fig. 3E). Plots of the fractional changes in responsive cells (neural activity index, NAI: percentage of neurons responding to S1/percentage of neurons responding to S4) of Cre-expressing and Cre-negative neurons in the two genotypes revealed that the Cre-expressing neurons in the *Fmr1*^cKO^ mice displayed a larger NAI than the neighboring Cre-negative neurons (Fig. 3F). Next, we quantified functional connectivity among the Cre-expressing neurons in WT and *Fmr1*^cKO^ mice. Statistical analysis revealed that compared with those of WT mice, the Cre-expressing V1 neurons of *Fmr1*^cKO^ mice exhibited significantly higher-order correlation probabilities, whereas low-order range probabilities remained indistinguishable (Fig. 3G). Above all, these observations indicate that the specific knockout of the *Fmr1* gene in V1 contributed to the visual deficits in these mice.

**Figure 4:**
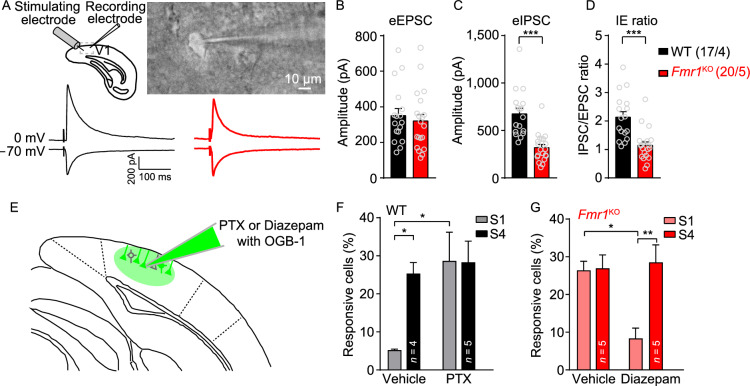
The GABAergic system in V1 in *Fmr1*^KO^ mice contributes to abnormal visual hypersensitivity. (A) Schematic representation of the recording layout (top left) and a bright-field image of an acute slice (top right). eEPSCs recorded at holding potential of −70 mV, and eIPSCs at 0 mV. Representative traces recorded from V1 L2/3 neurons of *Fmr1*^KO^ (bottom right) and WT (bottom left) littermates are also shown. (B–D) Quantification of eEPSC ((B) WT: 350.4 ± 39.24, *Fmr1*^KO^: 320.2 ± 37.06; *P* > 0.05) and eIPSC ((C), WT: 674.8 ± 60.14, *Fmr1*^KO^: 319.6 ± 32.78; *P* < 0.001) amplitude and the eIPSC/eEPSC (I/E) ratio ((D), WT: 2.14 ± 0.19, *Fmr1*^KO^: 1.14 ± 0.12; *P* < 0.001) of WT and *Fmr1*^KO^ mice. The values in parentheses indicate the number of neurons (left) and the number of mice (right) used in each experiment. (E) Schematic representation of the strategy for the local injection of picrotoxin (PTX, 100 μmol/L, 0.1 μL) or diazepam (100 μmol/L, 0.1 μL) with OGB-1, in the V1 of WT and *Fmr1*^KO^ mice. (F) Graph of average percentages (five trials, with each trial containing S1 and S4 in a random pattern) of responsive cells in the V1 of WT mice (vehicle, S1: 5.2% ± 2.2%; S4: 25.2% ± 2.8%, *P* < 0.05; PTX, S1: 28.5% ± 7.4%; S4: 28.1% ± 5.4%, *P* > 0.05). (G) Graph of average percentages (five trials, with each trial containing S1 and S4 in a random pattern) of responsive cells in the V1 of *Fmr1*^KO^ mice (vehicle: S1, 26.2% ± 2.3%, S4, 26.8% ± 3.4%, *P* > 0.5; diazepam: S1, 8.3% ± 2.6%, S4, 28.3% ± 4.5%, *P* < 0.01). Data are shown as the mean ± s.e.m. *P < 0.05, **P < 0.01, ***P < 0.001

To further confirm that V1 is essential for the visual processing abnormalities observed in *Fmr1*^KO^ mice, we examined the effect of manipulating the excitatory-inhibitory balance locally in V1 in mice at the same age as those described in Fig. 1. First, to determine the inhibitory/excitatory (I/E) ratio, we recorded evoked excitatory and inhibitory postsynaptic currents from the same V1 neurons in acute brain slices (Fig. 4A, top). While there was no change in the evoked excitatory postsynaptic currents (EPSCs) (Fig. 4B) in mutant V1 neurons, there was a significant decrease in the evoked inhibitory postsynaptic currents (IPSCs) (Fig. 4C). In addition, the I/E ratios were significantly decreased in mutant V1 neurons (Fig. 4D). Therefore, to block the inhibition of GABA receptors, we first locally injected the GABA_A_ receptor antagonist picrotoxin (Gibbs et al., 1997; Radzicki et al., 2017) (PTX) (Fig. 4E) to block inhibition and used vehicle (DMSO) as a control. Compared with vehicle treatment, PTX treatment dramatically increased the calcium response to low-intensity stimuli (Fig. 4F) in WT mice, resembling the phenotype observed in *Fmr1*^KO^ mice. Local administration of diazepam, a GABA_A_ receptor agonist (Ferreri et al., 2015; Ye et al., 2017), in the V1 of *Fmr1*^KO^ mice significantly reduced their visual hypersensitivity to low-intensity stimuli (Fig. 4G). Thus, local blockade of GABA receptors in V1 in WT mice is capable of mimicking the visual hypersensitivity observed in *Fmr1*^*KO*^ mice. Conversely, the activation of GABA receptors in V1 can reverse visual hypersensitivity in *Fmr1*^KO^ mice.

Together, these results demonstrate that the inactivation of *Fmr1* expression in V1 neurons was sufficient to induce enhanced responsiveness to low-intensity stimuli, which was accompanied by enhanced local functional connectivity independent of sensory inputs and lateral/upper modulations originating from the upstream brain regions.

### Rescue of visual hypersensitivity by restoring FMR1 in V1 neurons

**Figure 5:**
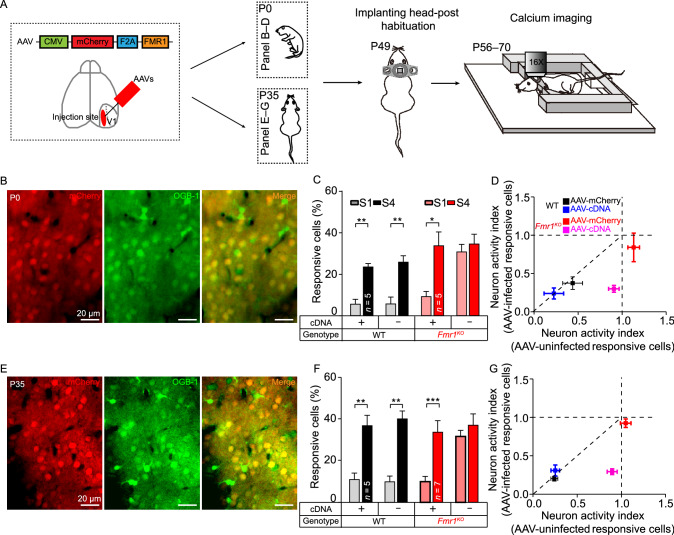
Overexpression of *Fmr1* cDNA in V1 neurons rescues visual hypersensitivity in *Fmr1*^KO^ mice. (A) Strategy of *Fmr1* cDNA overexpression in P0 and P35 mice. For P0 mouse injection, AAVs were injected at P0, the head-post was implanted at P49, and two-photon imaging was performed at P56-70. For P35 mouse injection, AAVs were injected at P35; the remaining of the steps were the same as those applied to P0 mice. (B) Representative average intensity projection of a representative calcium imaging movie (~5 s, 20.4 Hz) in L2/3 neurons infected with AAVs (red, injected at P0) and stained with OGB-1 (green). (C) Summary graph of the average percentages (five trials, with each trial containing S1 and S4 in a random pattern) of responsive AAV-uninfected (cDNA^-^) and AAV-infected (cDNA^+^) neurons in V1 to S1 and S4 stimuli in WT and *Fmr1*^KO^ mice infected with AAVs at P0 (WT^cDNA+^, S1: 6.01% ± 1.90%, S4: 24.04% ± 1.17%, *P* < 0.01; WT^cDNA-^, S1: 6.14% ± 2.9%, S4: 26.35% ± 2.67%, *P* < 0.01; *Fmr1*^KO; cDNA+^, S1: 9.70% ± 2.02%, S4: 34.35% ± 6.23%, *P* < 0.05; *Fmr1*^KO; cDNA-^, S1: 31.33% ± 3.26%, S4: 35.17% ± 4.31%, *P* > 0.05). (D) Plots of changes in the neuronal activity index (NAI) in AAV-infected and AAV-uninfected neurons in P0 WT and *Fmr1*^KO^ mice. (E) Representative average intensity stack of a representative calcium imaging movie (~5 s, 20.4 Hz) in L2/3 neurons infected with AAVs (red, injected at P35) and stained with OGB-1 (green). (F) Summary graph of the average percentages (five trials, with each trial containing S1 and S4 in a random pattern) of responsive AAV-infected (cDNA^+^) and AAV-uninfected (cDNA^-^) neurons in V1 to S1 and S4 stimuli in WT and *Fmr1*^KO^ mice infected with AAVs at P35 (WT^cDNA+^, S1: 11.50% ± 2.73%, S4: 37.64% ± 4.60%, *P* < 0.01; WT^cDNA-^, S1: 10.46% ± 2.30%, S4: 40.91% ± 3.42%, *P* < 0.01; *Fmr1*^KO; cDNA+^, S1: 10.51% ± 2.14%, S4: 34.48% ± 5.13%, *P* < 0.001; *Fmr1*^KO; cDNA-^, S1: 32.44% ± 2.49%, S4: 37.86% ± 5.08%, *P* > 0.05). (G) Plots of changes in the NAI in AAV-infected and AAV-uninfected neurons in P35 WT and *Fmr1*^KO^ mice. Data are shown as the mean ± s.e.m. *P < 0.05, **P < 0.01, ***P < 0.001

Restoring the function of a mutated gene is becoming an attractive therapeutic approach for the treatment of genetic disorders (Zeier et al., 2009; Gholizadeh et al., 2013; Kim et al., 2014), especially for diseases such as FXS that otherwise lack a cure (Arsenault et al., 2016). Based on our finding that the deletion of *Fmr1* specifically in V1 neurons is solely responsible for visual processing abnormalities, we tested whether reintroducing *Fmr1* into V1 neurons would rescue the enhanced responsiveness observed in *Fmr1*^KO^ mice. We stereotactically injected AAV-expressing mouse *Fmr1* cDNA (AAV9-CMV-mCherry-F2A-fmr1-WPRE-pA) with AAV-mCherry (as a control) into the V1 of P0 WT and *Fmr1*^KO^ mice (Fig. 5A, top). Two months after injection (the same age as the mice represented in Fig. 1), we loaded OGB-1 to perform *in vivo* calcium imaging (Fig. 5B) and compared the numbers of AAV-infected neurons and neighboring control neurons. In WT mice, expression of *Fmr1*^cDNA^ or mCherry did not alter the visual processing properties in V1 (Figs. 5C and S6A). Interestingly, in *Fmr1*^KO^ mice, we found that *Fmr1*^cDNA^-expressing neurons resembled WT neurons with regard to their input-output relationships to the visual stimulus; specifically, *Fmr1*^cDNA^-expressing (*Fmr1*^KO; cDNA^) neurons exhibited significantly less responsiveness to low-intensity stimuli than neighboring KO (*Fmr1*^KO^) neurons in *Fmr1*^KO^ mice (Fig. 5C). In addition, the expression of mCherry did not affect intensity-dependent visual processing in *Fmr1*^KO^ mice (Fig. S6A). Plots of the NAI of AAV-infected and AAV-uninfected neurons in the two genotypes show that the AAV-infected neurons in the *Fmr1*^KO^ mice displayed decreased NAI relative to the neighboring AAV-uninfected neurons (Fig. 5D). These results indicated that reintroducing *Fmr1* into V1 neurons of P0 *Fmr1*^KO^ mice could rescue the abnormal visual responsiveness.

A diagnosis of autism (and most neuropsychiatric disorders) usually occurs after the symptoms of the disease become apparent. Whether the damaged functions can be repaired after the physical connections of the neural microcircuits have formed is a critical clinical problem. Thus, we extended the time-point of gene restoration from P0 to P35 to test whether a “rescue-in-puberty” strategy could work for these vision-processing deficits (Fig. 5A, bottom and Fig. 5E). We showed that regardless of the kind of AAV (control or cDNA) injected, the response properties of the neurons in WT mice were not changed (Figs. 5F and S6B). However, the functional imaging results showed similar rescue effects with regard to the visual abnormalities observed in the neuronal activity of *Fmr1*^KO^ mice; specifically, we found that the percentage of low-intensity stimulus-activated neurons was significantly lower in *Fmr1*^KO; cDNA^ neurons than in neighboring *Fmr1*^KO^ neurons (Fig. 5F,* P* < 0.05). Similarly, the overexpression of a control AAV (AAV-mCherry) in V1 did not have a significant effect on the low-intensity stimulus-induced response in *Fmr1*^KO^ mice at P35 (Fig. S6B). In addition, plots of the NAI of AAV-infected and AAV-uninfected neurons in the two genotypes indicate that the AAV-infected neurons in the *Fmr1*^KO^ mice also displayed decreased NAI relative to the neighboring AAV-uninfected neurons (Fig. 5G). To confirm the rescue effect, we measured the I/E ratio of *Fmr1*^KO^ mice following overexpression of FMR1. Our results showed that the excitatory-inhibitory imbalance in the V1 region of *Fmr1*^KO^ mice can be rescued by the overexpression of FMR1 in *Fmr1*^KO^ mice at P0 (Fig. S7A and S7B) and P35 (Fig. S7C and S7D).

These results demonstrate that the vision abnormalities induced by the inactivation of *Fmr1* expression can be reversed by the restoration of FMR1 expression in either developing or developed V1 microcircuits, suggesting that there is an extended period in which potential treatments could be applied to restore visual deficits in FXS or autism.

## Discussion

Although progress in understanding of the molecular etiology of FXS has increased rapidly, an efficient cure is still lacking, especially in adult patients (Pop et al., 2014), which makes finding a rescue approach very important. The hyperexcitability and anxiety behaviors of FXS and ASD patients may be related to their hypersensitivity to sensory stimuli (Rais et al., 2018). In higher-severity ASD, local hyperconnectivity predominantly occurred in posterior brain regions, such as in regions that participate in visual processing (Keown et al., 2013). However, how these abnormalities affect behavior is still unknown. To fill this research gap, we performed two-photon imaging and implemented genetic approaches to explore visual processing deficits in *Fmr1*^KO^ mice. In this study, we obtained insights into the hypersensitivity to low-intensity visual stimuli of *Fmr1*^KO^ mice. We found that in *Fmr1*^KO^ mice, the V1 microcircuit exhibited an enhanced response to low-intensity stimuli, while their response to high-intensity stimuli remained unchanged. This change was mainly caused by the deletion of the *Fmr1* gene in V1. Moreover, restoration of *Fmr1* gene expression in *Fmr1*^KO^ mice, specifically in V1 neurons, rescued these visual processing abnormalities, even in mice with complete brain development (P35).

Changes in visual perception, especially the superior processing of local structure and fine detail, have been reported in FXS and autism patients (Cornish et al., 1999; Irwin et al., 2001; Scerif et al., 2004; Dakin and Frith 2005; Farzin et al., 2008; Rais et al., 2018). ASD patients have demonstrated increased performance in tasks requiring the recognition of details, the ability to find hidden figures (Shah and Frith 1983), and attention to small elements, such as in visual searches (Plaisted et al., 1998a; O’Riordan et al., 2001) and in the learning of highly confusing patterns (Plaisted et al., 1998b). Increased sensitivity to visual stimuli (gaze aversion) (Merenstein et al., 1996) and enhanced amplitude of visually evoked potentials (Knoth et al., 2014) are observed in FXS patients. In the present study, we found that *Fmr1*^KO^ mice were more sensitive to low-intensity stimuli, which is consistent with the performance of patients with FXS and ASD. The lower threshold of low-level vision perception observed in the *Fmr1*^KO^ mice does not seem to be restricted to visual intensity or vision perception alone. In a previous study, visual discrimination was also found to be impaired in *Fmr1*^KO^ mice, where it was correlated with marked deficits in the orientation tuning of principal neurons in V1 (Goel et al., 2018). Thus, the defect of V1 maybe a major inducing factor in the visual processing defect of the *Fmr1*^KO^ mice.

It is commonly believed that disrupted networks and connectivity across and within brain regions are a pathophysiological cause of FXS and autism. Functional magnetic resonance imaging (fMRI) and blood-oxygen-level-dependent (BOLD) studies suggest that patients with ASD and FXS exhibit weaker long-range brain connectivity and greater local functional connectivity than normally developing persons (Uddin et al., 2013; Haberl 2015). Additionally, many studies have indicated that there is a preference for local processing in ASD, which may suggest that visual perception is generally enhanced in ASD (Mottron et al., 2006; Keown et al., 2013). Our functional and anatomical analysis of V1 provides evidence for local hyperconnectivity in *Fmr1*^KO^ mice. The analysis of V1 microcircuits indicates that the enhanced connection of local networks is accompanied by an increase in the structural complexity of pyramidal neurons. This increase in structural complexity is consistent with previous reports showing an increase in the number of neuronal dendritic filopodia in this mouse model (Irwin et al., 2000; Kogan et al., 2008; Farzin et al., 2011; Berman et al., 2012; He and Portera-Cailliau 2013; Tang et al., 2014).

In *Fmr1*^KO^ mice, most previous studies have found that synaptic connections are attenuated or weakened because FMR1 negatively regulates the synapse number (Pfeiffer Brad and Huber, 2007, 2009; Ingrid et al., 2008). Few studies have explored the connectivity of brain networks at the single-neuron level in diseased brains. Through a synchronization analysis of neural networks, we found that the high-order correlations of neural networks were enhanced in *Fmr1*^KO^ animals, while the spike probability of single cells was not increased. These results support the theory that increased local connectivity might contribute to “islets of superior functioning” in the sensory (visual, auditory and tactile) neocortex in FXS (Rudie and Dapretto 2013) and the enhanced amplitudes of visually evoked potentials (Knoth et al., 2014) observed in FXS patients.

Subtle changes in brain development can cause intellectual and behavioral deficits. We found that the functional correlation and neural complexity in V1 are abnormally high, which predicted a significant impact on the ability of the brain to process sensory inputs and a negative impact on animal behavior. Approximately 90% of projections from the eye pass through the LGN to V1, which is uniquely positioned as the primary distributor of almost all visual information that reaches other cortical areas (Frank 2003). Our results indicate that the Cre-transfected neurons in the V1 region that cause the enhanced responsiveness to the S1 stimulus exhibit the abnormality observed in the *Fmr1*^KO^ mice, while the neighboring Cre-negative neurons behave normally. Combined with the results in the *Fmr1*^KO^ mice, gene expression in V1 can reverse the abnormal phenotype; furthermore, our results show that the elevated responsiveness to the S1 stimulus was cell-autonomous, suggesting that this phenotype may be triggered by the loss of FMR1 in single neurons. Above all, our results suggest that V1 is implicated as one of the dominant brain areas that contributes to visual sensory deficits in patients and may explain the superior processing in FXS and autism patients.

Our finding that visual processing abnormalities were recovered by the restoration of *Fmr1* expression in both newborn (P0) and juvenile (P35) V1 neurons in *Fmr1*^KO^ mice has important clinical implications and could lead to improved FXS therapies; for example, gene therapy could be a rapid and post-diagnostic therapy for FXS patients, especially as drugs developed to treat FXS have yet to be successful (Michalon et al., 2012; Dolan et al., 2013; Pop et al., 2014; Sun et al., 2016). In early life, before the acquisition of verbal language, infants initially rely heavily on touch and then on both touch and vision to sense the environment (Corbetta and Snapp-Childs 2009). Thus, in the early stages of development, relieving the visual (or other sensory) processing load for simple visual signals (such as the intensity of visual stimuli) may preserve the ability of the visual system to process complex signals related to social input and cognition, which may improve the corresponding ability in affected children (Colman et al., 1976; Coulter 2009; Aoki et al., 2019).

Sensory overload serves as one of the mechanisms underlying the social deficits observed in FXS and autistic patients (Rais et al., 2018). Gene therapy may have significant therapeutic effects on cognitive and other core symptoms in FXS and autism. Thus, in the early stages of development, relieving the visual (or other sensory) processor’s processing load on simple visual signals (such as the intensity of visual stimuli) is supposed to preserve the ability of the visual system to process complex signals related to social ability and cognition, which can be expected to have a significant effect on improving the corresponding ability of children. Our research provides a possible strategy to treat visual hypersensitivity in FXS and ASD.

## Methods

### Animals

All procedures were conducted in accordance with the Guidelines for the Care and Use of Laboratory Animals (8th edition) and approved by the Institutional Animal Care and Use Committee of Peking University. *Fmr1*^KO^ male mice (aged 2–3 months, FVB background) were purchased from Jackson Laboratory (Bar Harbor, Maine, USA, strain No: 003025), and *Fmr1*^cKO^ mice (aged 2–3 months, C57 background) were gifted from Prof. David L Nelson of the Baylor College of Medicine. All experiments were performed with WT male littermate controls and studied by experimenters who were blind to genotype. The animals were housed in rooms maintained on a 12-h light/12-h dark cycle with ad libitum access to water and food until acclimated to the imaging conditions. All experiments used littermate controls and were performed during the light cycle.

### Surgical preparation

This surgery was carried out as previously described (Tian et al., 2018). Briefly, healthy, young-adult mice (7 weeks old) were selected to undergo headpost-implantation surgery. The mice were anesthetized with an intraperitoneal injection of tribromoethanol (240 mg/kg, Sigma-Aldrich, St. Louis, Missouri, USA) and dexamethasone (5 mg/kg, Cisen Pharmaceutical Co., Ltd. Jining, Shandong, China). A metal adapter with a hole was attached to the skull using cyanoacrylate adhesive and dental acrylic cement (Nissin Dental Products Co., Ltd. Kunshan, Jiangsu, China). The location of V1 was determined using stereotactic coordinates (AP -2.8; ML 2.5; DV 0.2). The mice were allowed to recover for at least 7 days after surgery and were intraperitoneally administered ceftriaxone sodium (200 mg/kg, Youcare Pharmaceutical Group Co., Ltd. Beijing, China) daily to prevent inflammation. To familiarize the mice with the imaging device, each animal was placed in a head-restraint apparatus for 50–60 min every day for 3–5 days with the goal of reducing anxiety and movement during the actual procedure.

Before the imaging experiment, a square craniotomy of approximately 4 × 4 mm^2^ was carefully drilled into the right V1 while leaving the dura intact. A glass micropipette (WPI, Sarasota, Florida, USA) was fabricated using a P97 puller (Sutter) to create a resistance between 3 and 5 MΩ. Neurons were then labeled with the calcium indicator dye OGB-1 via bolus loading. OGB-1 (Life Technologies, Eugene, Oregon, USA) at a final working concentration of 0.5 mmol/L was prepared as described previously (Tian et al., 2018). To label cortical layer 2/3 (L2/3) neurons, the dye was pressure-injected through a micropipette inserted to a depth of 0.2–0.3 mm. The craniotomy was covered with a small piece of coverslip glass (0.13–0.17 mm thick, Diamond), and imaging commenced 1 h after dye injection.

### *In vivo* two-photon imaging

Fluorescence was monitored in L2/3 neurons in V1 at 920 nm using a commercial two-photon microscope (B-Scope, ThorLabs, Newton, New Jersey, USA) equipped with a tunable ultrafast laser (MaiTai BB DS-OL, Spectra-Physics, Santa Clara, USA) and a 16× water immersion objective (0.8 NA, Nikon, Tokyo, Japan). A 500–550 nm emission filter was used for dye detection. Scanning and image acquisition were controlled using ThorImageLS software (Thorlabs) at a sampling rate of 20.4 Hz. The size of the acquired images was 768 × 768 pixels from a field of view measuring 517.77 μm × 517.77 μm. The average power delivered to the brain was less than 70 mW. Every spontaneous recording lasted approximately 73.5 s, and each stimulus recording lasted 24.5 s. The focal plane and imaging position were checked and manually realigned with the initial image if necessary.

### Visual stimulation

The visual stimuli used to control stimulus drawing and timing were generated using custom software written in MATLAB (MathWorks, Natick, Massachusetts, USA) with the PsychToolbox extension (http://psychtoolbox.org). The display was positioned 20 cm directly in front of the mouse on a computer screen and centered to occupy 92° × 70.8° of the visual field (Fig. 1A). The visual stimulus consisted of one of four white spots (S1 = Φ70.8°, S2 = Φ39.1°, S3 = Φ20.2° and S4 = Φ10.2°), all of which were repeated 3 times in a random pattern, with each presented for 2 s (Fig. S2A).

### Fidgeting movement test behavior

A piezoelectric sensor was positioned under the forepaws of head-fixed mice as described previously (Cooke et al., 2015; Tian et al., 2018). Voltage signal changes indicating limb movements were recorded throughout the entire experiment at a sampling rate of 1000 Hz. For the vidget scores, the voltage signal (F) was down-sampled to 100 Hz, and the baseline (F_0_) was calculated by averaging the voltage signal throughout the entire session. The relative change in the voltage signal was calculated as (F−F_0_)/F_0_. To eliminate negative values, the extent of the vidget was calculated by taking the root mean square of the voltage signal (SQRT(X^2^)). The average value of the vidget score across 0.5 s after visual stimulation was used to quantify the degree of stimulus-driven movement and is presented in arbitrary units (a.u.).

### Virus injection

AAVs were used to locally infect V1 neurons. AAV9-hsyn-cre-mCherry-WPRE-pA was purchased from SunBio Pharmaceutical Technology (Shanghai) Corp., Ltd. AAV9-CMV-mCherry-F2A-fmr1-WPRE-pA and control AAVs were purchased from OBiO Technology (Shanghai) Corp., Ltd. A glass micropipette and a nanoinjection system (Reward) were used to deliver 500 nL of the virus at a depth of 300 µm into the cortex. To induce local deletion of *Fmr1*, V1 neurons in ~5-week-old mice were infected (Fig. 3A). In the *Fmr1* cDNA overexpression experiments, both P0 mice and ~5-week-old mice were infected (Fig. 5A).

### Morphological analysis

The three-dimensional neuroanatomical architecture of Golgi-stained whole mouse brains was obtained using a MOST system with a spatial resolution of 0.35 × 0.35 × 1 μm^3^. The tracing and reconstruction of dendrite morphology were conducted using Amira software (v5.2.2, FEI, Mérignac Cedex, France) and restricted to pyramidal and stellate-shaped neurons in a selected 1 × 1 × 1 mm^3^ cube (Figs. 2F, S5A and S5D). We visually inspected the dendritic branches and adjusted the position of the cube before tracing to ensure target neuron integrity. Morphology was analyzed using the Neurolucida Explore software (MBF Bioscience, Williston, VT, USA). The measured parameters included the number of primary dendrites, the number of nodes (branch points), the number of branch terminals, total dendrite length (μm), mean dendrite length (μm), and DC. DC was calculated according to the following equation: DC = [sum of the terminal orders + number of terminals] * [total dendritic length/number of primary dendrites].

### Electrophysiology

Whole-cell patch-clamp recordings were performed on V1 neurons from 2- to 3-month-old *Fmr1*^KO^ and littermate WT mice. Acute brain slices (400 mm) were prepared in an ice-cold dissection solution (213 mmol/L sucrose, 10 mmol/L glucose, 3 mmol/L KCl, 1 mmol/L NaH_2_PO_4_, 0.5 mmol/L CaCl_2_, 5 mmol/L MgCl_2_, and 26 mmol/L NaHCO_3_). The slices were maintained in a storage chamber containing artificial cerebrospinal fluid (aCSF) (10 mmol/L glucose, 125 mmol/L NaCl, 5 mmol/L KCl, 2 mmol/L NaH_2_PO_4_, 2.6 mmol/L CaCl_2_, 1.3 mmol/L MgCl_2_, and 26 mmol/L NaHCO_3_, pH 7.4, 300–310 mOsm) at room temperature. The aCSF was supplemented with a mixture of 95% O_2_–5% CO_2_ to maintain the pH at 7.4. Whole-cell recordings were performed using an EPC10 Patch-Clamp Amplifier (HEKA, Lambrecht, Germany) in V1 neurons. Patch pipettes (3–5 MΩ) were filled with internal solution consisting of 110 Cs-methanesulfonate, 20 TEA, 8 KCl, 10 HEPES, 10 EGTA, 5 QX-314, 3 Mg-ATP, 0.3 Na_2_GTP, with the pH adjusted to 7.2 and 305 Osm. The current was clamped at 0 pA for current-clamp recordings. EPSCs and IPSCs were evoked at holding potentials of −70 mV and 0 mV, respectively.

### Imaging data analysis

Imaging data were analyzed using ImageJ (National Institutes of Health, Bethesda, MD, USA) and semiautomatically digitized using a custom-made CNN-based program (Wang et al., 2019). Image sequences were first aligned for translational drift in ImageJ. The gray value of all pixels inside the cell body outline was then averaged to calculate the total intensity of the cell. The calcium signal was expressed as the change in relative fluorescence (ΔF/F_0_). For each cell, a transient signal was accepted when its intensity was greater than 2.5 times the standard deviation of the baseline. To quantify the activity of the visual cortical network, neurons with transient signals during the 2 s of visual stimulation were recorded as stimulus-responsive neurons. All traces were manually inspected after the automated analysis.

### High-order correlation

The calcium signals were translated into a binary time series in which a “1” indicated the peak of the original Ca^2+^ spike. The size of the neural circuit usually varies according to the experimental conditions, such as between different brain regions in the same mouse or within the same region among different mice. To generalize this analytical method, we randomly chose a subgroup with N neurons for all recorded neural circuits to calculate high-order correlations. The total point number (${Total}_{n}$) of a binary time series for each neuron was calculated by multiplying the recording time length (T) by the sampling frequency (f). The occurrence of simultaneous Ca^2+^ spikes in *n* neurons was defined as the *n*-order correlation, and the corresponding count was recorded as $Firing(n)$. The probability of an n-order correlation was calculated as ${Correlation}_{prob}\left(n\right)= Firing(n)/{Total}_{n}$. Independent correlations were evaluated using Monte Carlo methods after the neural binary time series was shuffled with T = 73.5 s, f = 20.4 Hz, N = 30.

### Location distribution patterns of neuron groups

All observed neurons were classified into different groups according to several criteria, such as whether they responded to specific stimuli. Each group of neurons was related to a specific functional neural assembly. The NND was unable to evaluate the location distribution patterns of these neural assemblies because the sizes of the neuron groups were usually unequal. To address this problem, we measured the NND in a random k-subgroup to describe the dispersion degree of these neuron groups (Fig. S3A). First, for each selected neuron in a given neuron group, we generated a random k-subgroup by randomly choosing k other neurons. Then, we calculated the NND between the selected neuron and the random k-subgroup. For each selected neuron, the random process was repeated until the NNDs converged.

### Analysis of pairwise correlation and the spatial structure of neuron populations

Pixels within the cell body were averaged to yield a time course (ΔF/F) for each neuron. The ΔF/F traces were low-pass-filtered using the Butterworth filter and then deconvolved with a 3-s exponential kernel. All points in the deconvolved traces that were below 2 times the standard deviation was set to zero. For $X=\{{x}_{1},{x}_{2},{x}_{3},\dots {,x}_{N}\}$ and $Y=\{{y}_{1},{y}_{2},{y}_{3},\dots {,y}_{N}\}$, which represent the deconvolved traces of a pair of neurons in vector form, the coefficient between the two neurons was defined as $Coefficient\left( {X,Y} \right) = A \cdot B/[EuclideanNorm\left( A \right) \times EuclideanNorm\left( A \right)]$. The Euclidean distance between each pair of neurons was also calculated to determine the spatial structure pattern of the neuron population. The coefficient correlation of the neurons was grouped based on the distance between the pair of neurons, and the correlation coefficient of each group was calculated.

### Statistical analysis

All statistical analyses were performed using the GraphPad Prism software (GraphPad Software, Inc., La Jolla, California, USA). Data are presented as the mean ± standard error of the mean (s.e.m.). Parametric statistical analyses were performed using Student’s t-tests, nonparametric tests were performed using Mann-Whitney tests, and comparisons across multiple groups were performed using the Kruskal-Wallis test. Significance levels are represented in the figures as follows: *P < 0.05, **P < 0.01, and ***P < 0.001.

## Supplementary Information

Below is the link to the electronic supplementary material.

Supplementary file1 (PDF 928 kb)

## Abbreviations

ASD: autism spectrum disorder
BOLD: blood-oxygen-level-dependent
*Fmr1*: fragile-X mental retardation 1
fMRI: functional magnetic resonance imaging
FMRP: fragile-X mental retardation protein
FXS: fragile X syndrome
GABA: γ-aminobutyric acid
LGN: lateral geniculate nucleus
MOST: micro-optical sectioning tomography
NAI: neural activity index
NND: nearest-neighbor-distance
OGB-1: oregon Green 488 BAPTA-1-AM
PFC: prefrontal cortex
PTX: picrotoxin
V1: primary visual cortex
WT: wild-type

## Declarations

This work was supported by grants from the National Basic Research Program of China (2017YFA0105201); the National Science Foundation of China (81925011 and 82001145); Key-Area Research and Development Program of Guangdong Province (2019B030335001); The Youth Beijing Scholars Program (015), Support Project of High-level Teachers in Beijing Municipal Universities (CIT&TCD20190334); and Beijing NSF Program and Scientific Research Key Program of Beijing Municipal Commission of Education (KZ201910025025, Z181100001518001); Chinese Institute for Brain Research, Beijing.

All institutional and national guidelines for the care and use of laboratory animals were followed.

The datasets in the study are available from the corresponding author on reasonable request.

C.Y., Y.T. and Y.C. carried out the experiments; C.Y., Y.T., F.S., Y.W., M.L., Y.W. and Y.C. performed the data analysis; P.Y., X.L., A.L., H.G., Q.L., D.Z., P.C., Y.L., X.W., M.L., W.X., L.C., X.-Y.L. and C.Z. contributed to the planning of the work; C.Z. wrote the manuscript.
