# Supplementary material for: Restoration of FMRP expression in adult V1 neurons rescues visual deficits in a mouse model of fragile X syndrome
Source: Protein Cell. 2021 Oct 29;13(3):203–19. doi: 10.1007/s13238-021-00878-z (PMC8901859; doi:10.1007/s13238-021-00878-z)
Supplement: Supplementary file 1 — Supplementary file1 (PDF 928 kb) [file 13238_2021_878_MOESM1_ESM.pdf]

Restoration of FMRP Expression in Adult V1 Neurons Rescues Visual Deficits in a Mouse Model of Fragile X Syndrome

Supplemental Information

Supplementary Figures

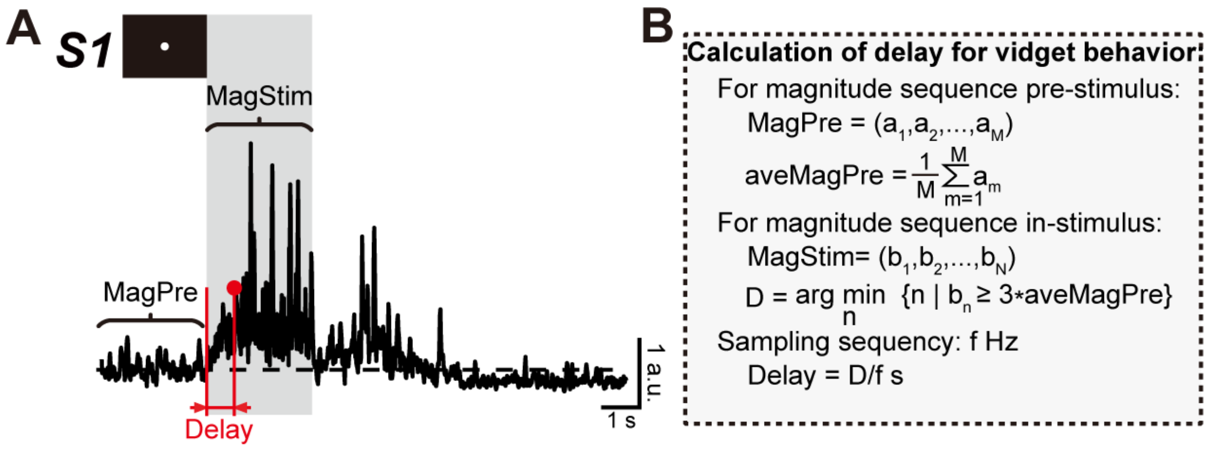

Supplementary Fig. 1 | Calculation of the delay in vidget behavior.

A. Vidget trace of a visual-stimulus-induced neuron. The gray area indicates the stimulus period (2 s). MagPre and MagStim denote the vidget magnitude during the prestimulus and in-stimulus periods, respectively. The dashed line denotes the average value of the magnitude during the prestimulus period (aveMagPre).

B. mathematical definition and calculation of delay based on the vidget trace.

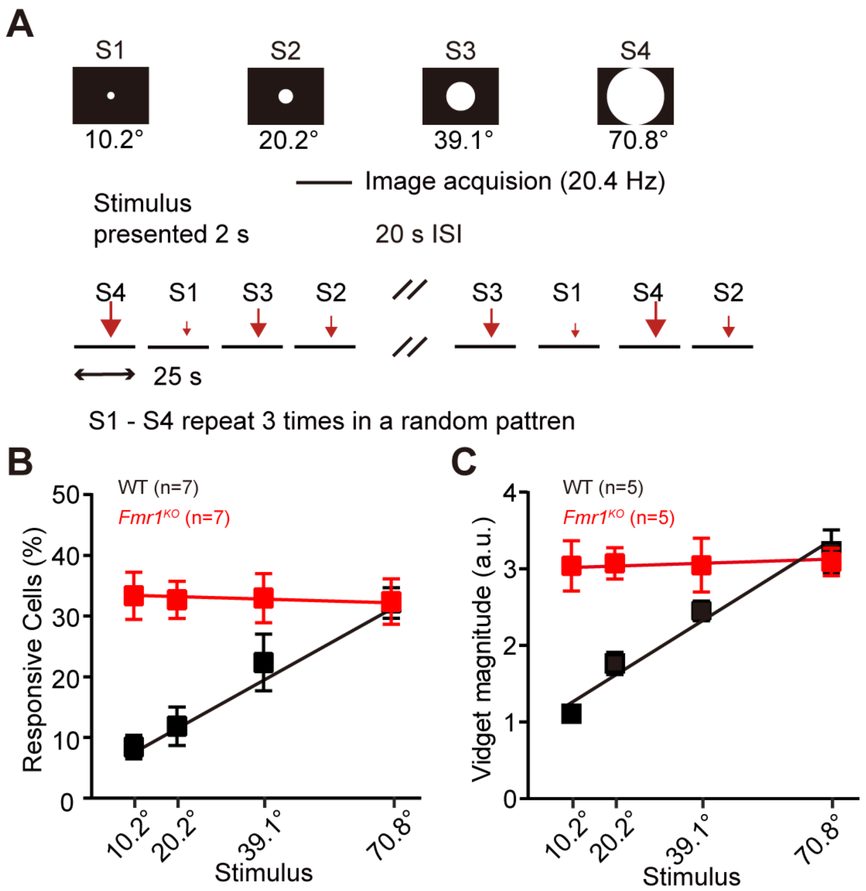

Supplementary Fig. 2 | Schematic representation of the stimulus strategy.

A. The visual stimuli S1, S2, S3, and S4 are presented as white circles that occupy 10.2°, 20.2°, 39.1°, and 70.8° of the visual field, respectively. All four stimuli were repeated 3 times in random patterns. Each stimulus was presented for 2 s, and the stimuli were separated by a 20-s black screen.

B and C. Average visual stimulus-induced neuronal (slope, wild-type (WT): 0.39 ± 0.03, *Fmr1*^{KO}: 0.01 ± 0.01, two-way ANOVA, $p < 0.01$) and behavioral (slope, WT: (3.38 ± 0.48) ×10^{-2}, *Fmr1*^{KO}: (0.06 ± 0.05 ×10-2, two-way ANOVA, $p < 0.01$) responses of WT (black) and *Fmr1*^{KO} (red) mice over 3 experimental trials (each of which presented S1/2/3/4 in a random pattern).

Data are shown as the mean ± s.e.m.

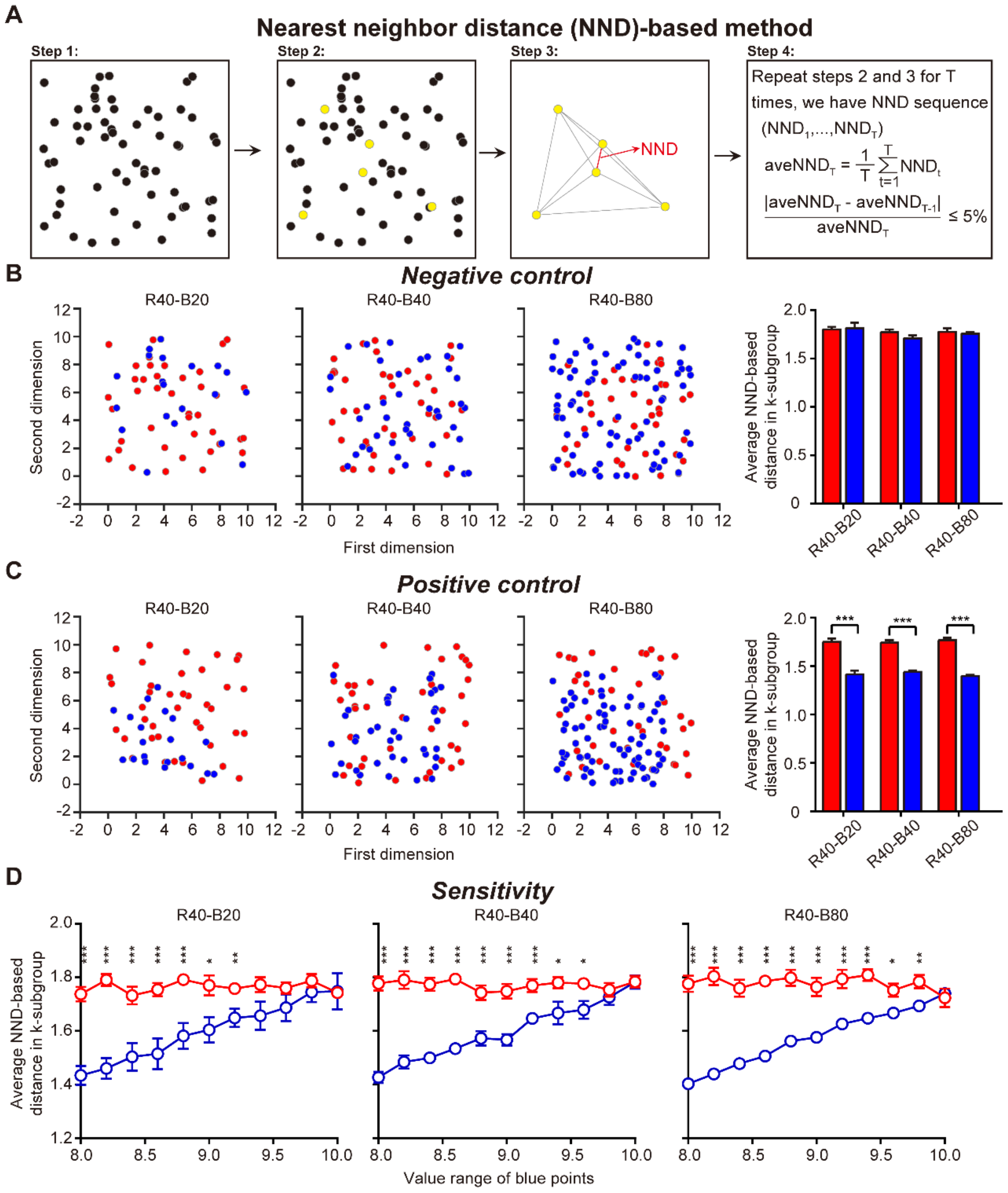

Supplementary Fig. 3 | Implementation and reliability test of the nearest neighbor distance (NND)-based method.

A. Diagram of the implementation of the NND-based method. Step 1: Initialize the spatial position for all points (black circles). Step 2: Randomly choose the k-subgroup of points (yellow circles, k=10). Step 3: Calculate the pairwise distances among the selected k-subgroup and find the nearest-neighbor distance (NND). Step 4: Repeat steps 2 and 3 until the average NND converges. Note that the algorithm stops if all possible combinations of k-subgroups are exhausted even if the NND does not converge.

B. Negative control experiments for the NND-based method. Left, two-dimensional red and blue points were randomly generated. The value ranges for the red dots and blue dots were [0 10] and showed a uniform distribution. The number of the red dots was 40 (R40). The number of the blue dots were 20 (B20), 40 (B40), and 80 (B80). Right, summary graph of NND-based distances for red and blue points in the k-subgroup (k=10) for the three-point groups (R40-B20, R40-B40, and R40-B80).

C. Positive control experiments for the NND-based method. The value ranges of the red dots and blue dots were [0 10] and [0 8], respectively.

D. Sensitivity test for the NND-based method. The value range of the red dots was [0 10]. The value range of the blue dots was dynamic [0 N], with N changing from 8 to 10. The plot of the average NND-based distance versus the value range of the blue dots is shown for the three-point groups (R40-B20, R40-B40, and R40-B80).

Data are shown as the mean ± s.e.m. *$p < 0.05$, **$p < 0.01$, ***$p < 0.001$.

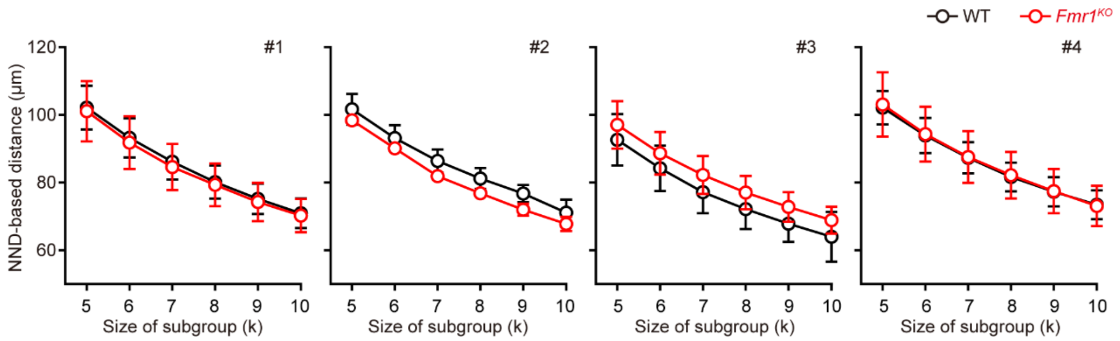

Supplementary Fig. 4 | Plot of NND-based distance for the four types of neurons versus the sizes of the subgroups.

A random k-subgroup containing 5 to 10 neurons was considered. WT, black. *Fmr1*^{KO}, red. Data are shown as the mean ± s.e.m. Type #1, neurons responded specifically to S4. Type #2, neurons responded specifically to S1. Type #3, neurons responded to both S4 and S1. Type #4, neurons did not respond to either stimulus.

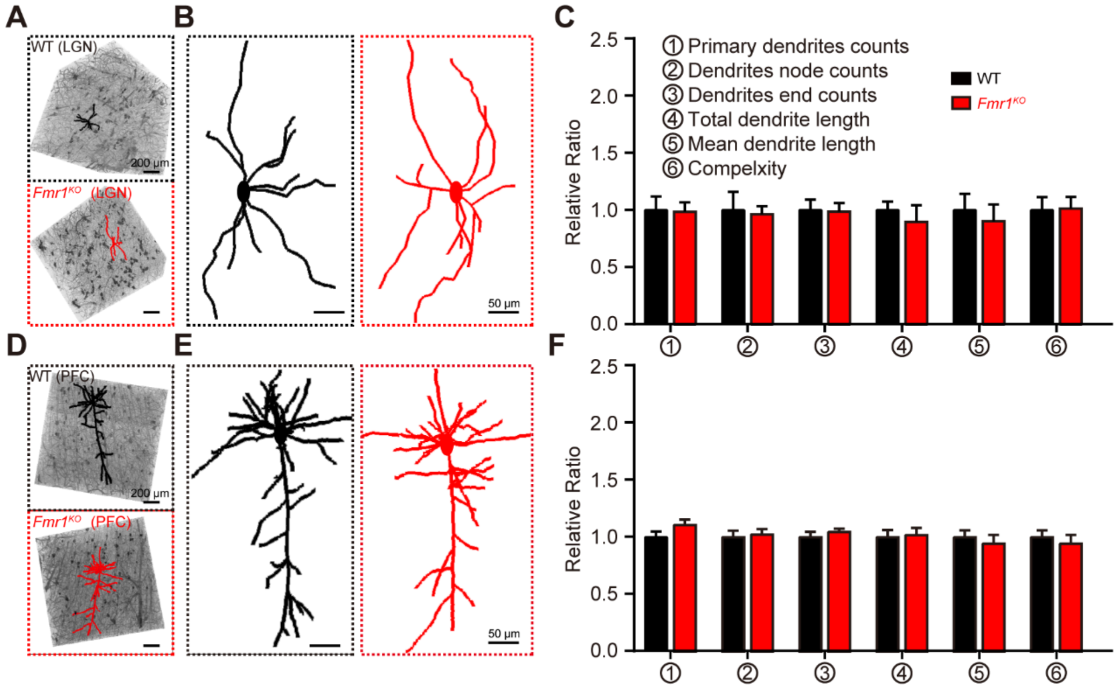

Supplementary Fig. 5 | Neuronal morphology of lateral geniculate nucleus (LGN) and prefrontal cortex (PFC) neurons in WT and *Fmr1^{KO}* mice.

A. 3D plot of the reconstructed morphologies of LGN neurons in WT (upper) and *Fmr1^{KO}* (bottom) mice.

B. 2D plot of the reconstructed morphologies of LGN neurons in WT (left) and *Fmr1^{KO}* (right) mice.

C. Summary graph of the relative proportions of primary dendrite counts (WT: 1.00 ± 0.45; *Fmr1^{KO}*: 0.98 ± 0.37, $p > 0.05$), dendrite node counts (WT: 1.00 ± 0.28; *Fmr1^{KO}*: 0.90 ± 0.63, $p < 0.05$), dendrite end counts (WT: 1.00 ± 0.56; *Fmr1^{KO}*: 0.90 ± 0.65, $p > 0.05$), total dendrite lengths (WT: 1.00 ± 0.60; *Fmr1^{KO}*: 0.96 ± 0.28, $p > 0.05$), mean dendrite lengths (WT: 1.00 ± 0.32; *Fmr1^{KO}*: 0.99 ± 0.33, $p > 0.05$), and complexity (WT: 1.00 ± 0.38; *Fmr1^{KO}*: 1.01 ± 0.38, $p > 0.05$) of LGN neurons. All six measurements were normalized to the corresponding average value obtained in the WT mice.

D. 3D plot of the reconstructed morphologies of PFC neurons in WT (upper) and *Fmr1^{KO}* (bottom) mice.

E. 2D plot of the reconstructed morphologies of PFC neurons in WT (left) and *Fmr1^{KO}* (right) mice.

F. Summary graph of the relative ratios of primary dendrite counts (WT: 1.00 ± 0.17; *Fmr1^{KO}*: 1.11 ± 0.17, $p > 0.05$), dendrite node counts (WT: 1.00 ± 0.22; *Fmr1^{KO}*: 1.02 ± 0.22, $p > 0.05$), dendrite end counts (WT: 1.00 ± 0.20; *Fmr1^{KO}*: 0.94 ± 0.27, $p > 0.05$), total dendrite lengths (WT: 1.00 ± 0.21; *Fmr1^{KO}*: 1.02 ± 0.17, $p > 0.05$), mean dendrite

lengths (WT: 1.00 ± 0.17; *Fmr1*^{KO}: 1.05 ± 0.10, $p > 0.05$), and complexity (WT: 1.00 ± 0.17; *Fmr1*^{KO}: 0.93 ± 0.12, $p > 0.05$) of PFC neurons.

Data are shown as the mean ± s.e.m.

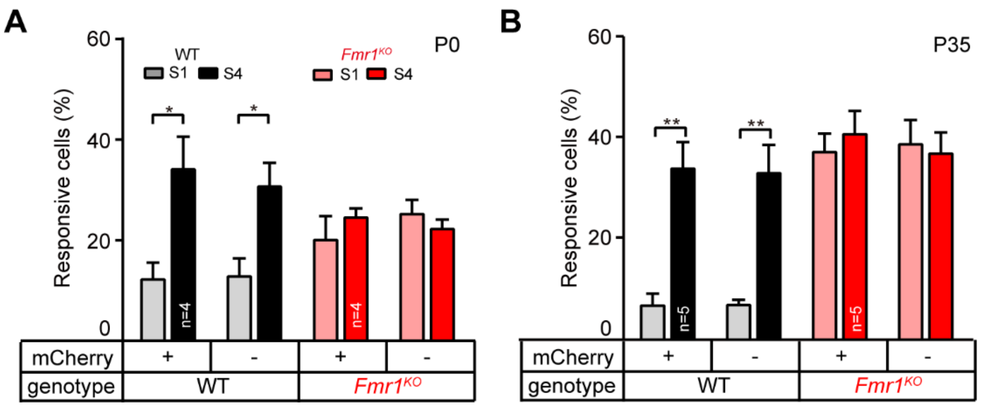

Supplementary Fig. 6 | Injection of control adeno-associated viruses (AAVs) into V1 does not affect the phenotypes of WT and *Fmr1*^{KO} mice.

A. Graph of average percentages (five trials, with each trial presenting S1 and S4 in a random pattern) of responsive AAV-uninfected (mCherry$^-$) and AAV-infected (mCherry$^+$) neurons in V1 to S1 and S4 stimuli in WT (n=5) and *Fmr1*^{KO} mice (n=5) infected with control AAVs at P0 (WT$^{mCherry+}$, S1: 12.74 ± 2.90%, S4: 34.71 ± 6.12%, $p < 0.05$; WT$^{mCherry-}$, S1: 13.34 ± 3.14%, S4: 31.29 ± 4.31%, $p < 0.05$; *Fmr1*$^{KO;\ mCherry+}$, S1: 20.61 ± 4.30%, S4: 25.05 ± 1.44%, $p > 0.05$; *Fmr1*$^{KO;\ mCherry-}$, S1: 25.78 ± 2.37%, S4: 22.78 ± 1.44%, $p > 0.05$).

B. Graph of average percentages (five trials, with each trial presenting S1 and S4 in a random pattern) of responsive AAV-infected (mCherry+) and AAV-uninfected (mCherry-) neurons in V1 to S1 and S4 stimuli in P35 WT and *Fmr1*^{KO} mice infected with control AAVs at P35 (WT$^{mCherry+}$, S1: 7.27 ± 1.95% S4: 34.63 ± 4.84%, $p < 0.01$; WT$^{mCherry-}$, S1: 7.40 ± 0.56%, S4: 33.73 ± 5.20%, $p < 0.01$; *Fmr1*$^{KO;\ mCherry+}$, S1: 37.91 ± 3,27%, S4: 41.52 ± 4.20%, $p > 0.05$; *Fmr1*$^{KO;\ mCherry-}$, 39.48 ± 4.46%, S4: 37.65 ± 3.76%, $p > 0.05$).

Data are shown as the mean ± s.e.m. *$p < 0.05$, **$p < 0.01$.

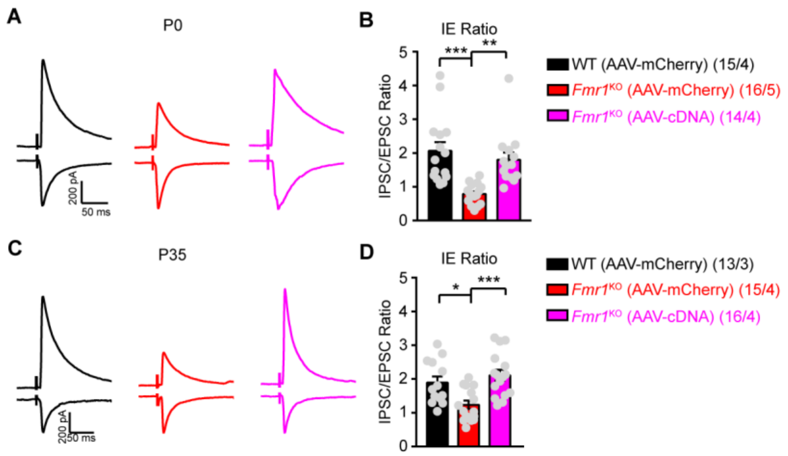

Supplementary Fig. 7 | Overexpression of FMRP at P0 and P35 increases the evoked inhibitory postsynaptic current/evoked excitatory postsynaptic current (eIPSC/eEPSC) ratio in the V1 neurons of *Fmr1*^{KO} mice.

A. Representative traces of eEPSCs and eIPSCs recorded from V1 neurons after AAV injection at P0.

B. Quantification of the eIPSC/eEPSC ratio of WT and *Fmr1*^{KO} mice after AAV-mCherry or AAV-cDNA injection at P0 (WT$^{mCherry+}$: 2.06 ± 0.26, *Fmr1*$^{KO;\ mCherry+}$: 0.79 ± 0.07, *Fmr1*$^{KO;\ cDNA+}$: 1.80 ± 0.21). The numbers in parentheses represents the number of V1 neurons (left) and the number of mice (right) used in the experiment.

C. Representative traces of eEPSCs and eIPSCs recorded from V1 neurons after AAV injection at P35. The numbers in parentheses represents the number of V1 neurons (left) and the number of mice (right) used in the experiment.

D. Quantification of the eIPSC/eEPSC ratio of WT and *Fmr1*^{KO} mice after AAV-mCherry or AAV-cDNA injection at P35 (WT$^{mCherry+}$: 1.89 ± 0.18, *Fmr1*$^{KO;\ mCherry+}$: 1.23 ± 0.12, *Fmr1*$^{KO;\ cDNA+}$: 2.11 ± 0.16).

Data are shown as the mean ± s.e.m. $*p < 0.05$, $**p < 0.01$, $***p < 0.001$.
